# Fructose Induces Visceral Adipose Tissue Inflammation and Insulin Resistance Even Without Development of Obesity in Adult Female but Not in Male Rats

**DOI:** 10.3389/fnut.2021.749328

**Published:** 2021-11-11

**Authors:** Sanja Kovačević, Jelena Brkljačić, Danijela Vojnović Milutinović, Ljupka Gligorovska, Biljana Bursać, Ivana Elaković, Ana Djordjevic

**Affiliations:** Department of Biochemistry, Institute for Biological Research “Siniša Stanković”-National Institute of Republic of Serbia, University of Belgrade, Belgrade, Serbia

**Keywords:** fructose diet, inflammation, visceral adipose tissue, insulin resistance, female rats

## Abstract

**Introduction:** Obesity and related metabolic disturbances are frequently related to modern lifestyle and are characterized by excessive fructose intake. Visceral adipose tissue (VAT) inflammation has a central role in the development of insulin resistance, type 2 diabetes (T2D), and metabolic syndrome. Since sex-related differences in susceptibility and progression of metabolic disorders are not yet fully understood, our aim was to examine inflammation and insulin signaling in VAT of fructose-fed female and male adult rats.

**Methods:** We analyzed effects of 9-week 10% fructose-enriched diet on energy intake, VAT mass and histology, and systemic insulin sensitivity. VAT insulin signaling and markers of VAT inflammation, and antioxidative defense status were also evaluated.

**Results:** The fructose diet had no effect on VAT mass and systemic insulin signaling in the female and male rats, while it raised plasma uric acid, increased PPARγ level in the VAT, and initiated the development of a distinctive population of small adipocytes in the females. Also, adipose tissue insulin resistance, evidenced by increased PTP1B and insulin receptor substrate 1 (IRS1) inhibitory phosphorylation and decreased Akt activity, was detected. In addition, fructose stimulated the nuclear accumulation of NFκB, increased expression of proinflammatory cytokines (IL-1β, IL-6, and TNFα), and protein level of macrophage marker F4/80, superoxide dismutase 1, and glutathione reductase. In contrast to the females, the fructose diet had no effect on plasma uric acid and VAT inflammation in the male rats, but less prominent alterations in VAT insulin signaling were observed.

**Conclusion:** Even though dietary fructose did not elicit changes in energy intake and led to obesity in the females, it initiated the proliferation of small-sized adipocytes capable of storing fats further. In contrast to the males, this state of VAT was accompanied with enhanced inflammation, which most likely contributed to the development of insulin resistance. The observed distinction could possibly originate from sex-related differences in uric acid metabolism. Our results suggest that VAT inflammation could precede obesity and start even before the measurable increase in VAT mass, making it a silent risk factor for the development of T2D. Our results emphasize that adipose tissue dysfunction, rather than its simple enlargement, could significantly contribute to the onset and development of obesity and related metabolic disorders.

## Introduction

Increased sugar consumption represents one of the major characteristics of modern way of living. Fructose, a tremendously abundant component of the modern diet, represents one of the major etiological factors in the development of metabolic disorders (1–3), because its intake has been associated with visceral adiposity, obesity, dyslipidemia, and type 2 diabetes (T2D) in both humans and animal models (4, 5). Although epidemiological studies suggest that global gender disparities exist in relation to obesity (6), the majority of animal and clinical research on metabolic disorders has been undertaken in males. The underrepresentation of female subjects in animal research has been increasingly recognized and even incorporated in the official guidelines (7, 8), since animal models created based on a single sex have resulted in shortcoming in the determination of whether obtained results are applicable to both sexes. Furthermore, studies referring to sex-specific differences in energy homeostasis and metabolic dysfunction could enable the future development of relevant sex-based therapeutic strategies for diabetes, metabolic syndrome, and obesity (9).

An excessive amount of adipose tissue, and, specifically, its accumulation in the abdominal region, have been recognized as major factors for adverse metabolic consequences of obesity, such as insulin resistance and development of T2D (10). The state of insulin resistance in the adipose tissue could be the result of change in abundance and affinity of insulin receptor (IR), as well as of altered posttranslational modifications of insulin receptor substrate 1 (IRS1), where its phosphorylation at serine 307 is considered inhibitory and generally impairs insulin signaling (11). Other important downstream mediators of tissue insulin action are phosphatidylinositol (PI) 3-kinase and Akt, the activation of which mediates insulin stimulation of glucose uptake and various other effects of insulin, such as inhibition of lipolysis and activation of fatty acid, glycogen, and protein, and DNA synthesis in the adipose tissue (12). Insulin action in tissues can also be modulated by a negative regulator, protein tyrosine phosphatase 1B (PTP1B), which dephosphorylates IR and its substrates, and its reduction regulates adiposity and the expression of genes involved in lipogenesis, such as peroxisome proliferator-activated receptor γ (PPARγ) (13). Fructose feeding has been found to specifically impair the activation of insulin signaling components, such as IR, IRS1, Akt, and PTP1B in the visceral adipose tissue (VAT) (14, 15). Insulin resistance in this particular depot is still considered an important factor in the development of systemic insulin resistance, mainly through the release of excess adipose tissue free fatty acids (FFAs) into the bloodstream (16).

It was shown more than two decades ago that insulin resistance in obesity was closely related to adipose tissue inflammation (17), when increased tumor necrosis factor α (TNFα) expression in the adipose tissue of obese rodents and humans was identified (18). Further animal and human studies confirmed the increased expression and/or secretion of several proinflammatory cytokines, such as TNFα, interleukin 1β (IL-1β) and interleukin 6 (IL-6) in the adipose tissue from obese subjects (19–21). According to the proposed mechanisms of the development of adipose tissue inflammation, hypertrophic adipocytes initially begin to secrete low levels of TNFα, which then stimulates the production of chemoattractant proteins capable of attracting macrophages to infiltrate into the adipose tissue (22, 23). Another presumption is that the death of lipid-engorged adipocytes stimulates the macrophage infiltration of the adipose tissue in obese rodents and humans (24). Obesity also causes a phenotypic switch in macrophage activation and polarization to classically activated M1 macrophages and alternatively activated, anti-inflammatory M2 phenotype (25, 26). Among surface markers of adipose tissue macrophages, the ones with high expression of F4/80 have been shown to produce more TNFα (27). The accumulation of macrophages in the adipose tissue drives a vicious cycle of their infiltration and further production of proinflammatory cytokines, often through the nuclear factor kappa B (NFκB) signaling pathway (28). Namely, TNFα activates NFκB transcriptional regulator, which in turn, upon activation, enters the nucleus and induces transcription of genes for TNFα, IL-6, and IL-1β (29). The molecular mechanisms behind inflammation-induced insulin resistance in the adipose tissue mainly rely on the findings that inflammatory cytokines, specifically TNFα, can activate c-Jun NH2-terminal kinase (JNK) and IKK serine-kinases, which promote the inhibitory phosphorylation of IRS1 on Ser^307^, leading to the disruption of insulin receptor signaling (30). Similar to TNFα, IL-1β was shown to reduce IRS1 expression at both transcriptional and posttranscriptional levels (31), while the IL-1β-mediated deterioration of insulin signaling is largely due to the IL-6 production and suppressor of cytokine signaling 3 (SOCS3) induction in 3T3-L1 adipocytes (32). In addition, the TNFα-induced expression of PTP1B, which dephosphorylates tyrosine residues on IRS1 making it less active, has also been shown in cultured human adipocytes (31).

It has been shown that systemic oxidative stress is related with the development of metabolic syndrome (33). Moreover, increased markers of oxidative stress have been positively correlated with higher body mass index (BMI) (34). In addition to the excessive production of reactive oxygen species (ROS), obese humans and animals also exhibit higher hydrogen peroxide production and reduced activities of antioxidant enzymes, such as superoxide dismutases 1 and 2 (SOD1 and SOD2), glutathione reductase (GRed), glutathione peroxidase (GPx), and catalase (CAT) in the adipose tissue (35–37). Oxidative stress was also found not only to correlate with adipose tissue insulin resistance, but to be a causative factor in its development (38). Namely, excessive ROS production can lead to the direct deregulation of insulin signaling by impairing inhibitory IRS1 phosphorylation (39), or can act indirectly through the NFκB activation and enhancement of proinflammatory cytokines (35). Also, fructose was reported to produce a pro-oxidative effect and to alter the expression of antioxidative enzymes in rats (40).

Sex-related differences in susceptibility and progression of metabolic disorders have gained much attention recently but are not yet fully understood. Studies on humans have shown that, although both males and females are susceptible to the effect of excess body fat on lipid and carbohydrate metabolism, T2D was more prevalent in men than in women and associated with a larger amount of VAT (41). Another study showed that, particularly in women, VAT was associated with insulin resistance and insulin secretion, and that in men, both VAT and SAT were associated with insulin resistance to a similar extent (42). Finally, sex-specific changes were also described after weight loss and in chronic low-grade inflammation specifically related to the epicardial fat depot, which is increasingly recognized as a metabolically active organ (43). Our previous studies on animal models have shown that male and female rats apply different strategies to cope with energy overload originating from fructose. While a fructose-rich diet applied immediately after weaning induced visceral adiposity in female rats (44), the same diet stimulated VAT lipolysis and led to elevated free fatty acid levels in males (45).

However, even within the same sex, immature young organisms and adult ones differ largely by their metabolic and physiological profiles (46), specifically in the capacity of VAT expansion, parameters related to fat accumulation (adiposity index and relative weights of different VAT depots), and adiponectin profile and leptin sensitivity (47, 48). We have previously shown that a fructose diet given to post-weaning female rats led to increased energy intake, higher VAT mass and VAT-body-ratio, and reduced blood glucose (49). Therefore, in this study, we sought to investigate the effects of the same dietary regime on the metabolic status of VAT in young adult females, which were 2.5 months old at the beginning of the treatment. Taking into account that VAT inflammation plays a central role in the development of insulin resistance and obesity-associated metabolic disturbances under burden of excessive sugar consumption, our aim was to examine the contributory role of VAT inflammation in the development of insulin resistance and obesity. This will be investigated and compared between female and male rats on fructose-enriched diet.

## Materials and Methods

### Material

Fructose was purchased from Apipek (Bečej, Serbia) and commercial rodent food from Veterinary Institute Subotica, Serbia. Anti-SOD1 (ab13498), anti-SOD2 (ab13533), anti-GRed (ab16801), anti-catalase (ab16731) and anti-GPx (ab22604) primary antibodies, and secondary anti-mouse (ab97046), anti-rabbit (ab6721) and anti-goat (ab6741) IgG H and L horseradish peroxidase (HRP)-linked antibody were obtained from Abcam (Cambridge, United Kingdom); anti-NFκB/p65 (sc-372), anti-phospho-Akt 1/2/3 (Ser473, sc-7985-R), anti-Akt 1/2/3 (sc-8312), anti-phospho-IRS-1 (Ser307, sc-33956), anti-IRS1 (E-12; sc-8038), anti-PTP1B (N-19, sc-1718-R), anti-lamin B (M-20; sc-6217), anti-F4/80 (sc-26643-R), anti-PPARγ antibody (sc-7273) were purchased from Santa Cruz Biotechnology (Dallas, TX, United States), and anti-β-actin antibody (AC 15) was purchased from Sigma Chemicals (St. Louis, MO, United States). The immobilon-FL polyvinylidene difluoride (PVDF) membrane was a product of Millipore (United States), while Amersham ECL Western Blotting Detection Kit was acquired from GE Healthcare Life Sciences. High-capacity cDNA reverse transcription kit, RNase inhibitor, TaqMan^®^ Universal PCR Master Mix with AmpErase UNG, and TaqMan^®^ Gene Expression Assay primer-probe mix for IL-1β (Rn00580432_m1), IL-6 (Rn01410330_m1), TNFα (Rn01525859_g1), and hypoxanthine phosphoribosyl transferase 1 (HPRT1) (Rn01527840_m1) were all products of Applied Biosystems. TRIzol^®^ Reagent (Ambion), RNase free DNase I (Ferments), and RNase-DNase free water (Eppendorf) were also used.

### Animals and Treatment

Female and male Wistar rats (2.5 months old), bred in our laboratory, were randomly divided in two experimental groups (*n* = 8–9 animals per group): a control group fed with commercial standard rodent food and drinking water and a fructose group fed with the same food and 10% (w/v) fructose solution instead of drinking water. Allocation of the animals to the experimental groups was performed by appropriate randomization method in order to ensure blinding and reduction of systematic differences in the characteristics of animals assigned to the experimental groups. All the experimental groups had *ad libitum* access to food and drinking fluid during the 9 weeks. Fructose concentration was chosen to resemble modern human lifestyle (50). Detailed composition of the food has been published previously (51). The animals were housed three per cage and kept under standard conditions at 22°C with a 12-h light/dark cycle and had constant veterinary care during the course of the experiment. Food and fluid intake per cage were recorded daily, and daily energy intake was calculated as follows: for control rats as calories ingested as food [food weight (g) × 11 kJ], while for fructose-fed rats was as sum of calories ingested as food and fructose solution [food weight (g) × 11 kJ + fructose intake (ml) × 1.72 kJ]. Body mass was recorded weekly. The study was conducted according to the guidelines from the EEC Directive 2010/63/EU on the protection of animals used for experimental and other scientific purposes, and was approved by the Ethical Committee for the Use of Laboratory Animals of the Institute for Biological Research “Siniša Stanković,” University of Belgrade (Permit No. 02-11/14 obtained on November 13, 2014).

### Plasma Parameters

The animals were killed by rapid decapitation after overnight fasting, during which all the experimental groups were provided with only drinking water. Vaginal smears were performed to determine the estrus cycle of the female rats; hence, all the female animals were killed in the diestrus phase of estrous cycle. Blood glucose levels were determined by MultiCare strips (Biochemical Systems International, Italy). After decapitation, the trunk blood from each experimental animal was collected in a separate tube with EDTA and centrifuged at 3,000 rpm for 10 min and supernatants were used as plasma and stored at −20°C until use. The level of plasma insulin was determined by the RIA method, using rat insulin standards (INEP, Serbia). Assay sensitivity was 0.6 mIU/L, and intra assay coefficient of variation was 5.24 %. Plasma uric acid concentration was determined commercially.

### Quantification of Insulin Sensitivity/Resistance

Insulin sensitivity was evaluated by homeostasis model assessment (HOMA) index calculation and intraperitoneal glucose tolerance test (IPGTT). The HOMA index was calculated from fasted plasma insulin and glucose concentration using the formula insulin (mU/L) × [glucose (mmol/L)/22.5]. IPGTT was performed 3 days before the end of the experimental period. Food was removed the night before, and the fructose solution was temporarily replaced with water. A glucose challenge (2 g/kg) was given intraperitoneally. Plasma glucose concentration was determined from the blood in the tail vein 0, 15, 30, 60, 90, and 120 min after the challenge injection. The area under the concentration vs. time curve (AUC glucose 0–120 min, mmol/L vs. min) was calculated by the trapezoidal rule.

### Tissue Preparation

Visceral (retroperitoneal, perirenal, and omental) adipose tissue was excised, washed with saline, dried, and stored in liquid nitrogen until use. After thawing, the tissue was homogenized in 1 vol (w/v) of ice-cold homogenization buffer (20 mMTris–HCl, pH 7.4, containing 10% glycerol, 50 mM NaCl, 2 mM dithiothreitol, 1 mM EDTA-Na_2_, 1 mM EGTA-Na_2_, 20 mM Na_2_MoO_4_, and protease and phosphatase inhibitors). A part of the homogenized tissue was sonicated 3 × 15 s on ice at 1A and 50/60 Hz, with 30% amplitude (Hielscher Ultrasound Processor) and centrifuged for 60 min at 105,000 g, 4°C. The supernatant was used as the whole cell extract. The rest of the homogenate was centrifuged for 10 min at 2,000g, 4°C, and the supernatant (S1) was used to obtain cytosol, while nucleosol was obtained from the pellet (P1). Supernatant S1 was centrifuged for 1 h at 105,000g, 4°C, and the final supernatant was used as the cytosol (52). To obtain nucleosol, pellet P1 was washed in.5 ml homogenization buffer (10 min at 2,000g, 4°C), resuspended in 1 vol (w/v) of NUN buffer (25 mM HEPES, pH 7.6, 1 M Urea, 300 mM NaCl, 1 % Nonidet P-40, 2 mM dithiothreitol, 20 mM Na_2_MoO_4_, and protease and phosphatase inhibitors), and incubated for 1 h in an ice bath with frequent vortexing. After centrifugation (10 min at 8,000g, 4°C), the supernatant was used as the nucleosol (53).

### Histological and Morphometric Analyses

After excision, the adipose tissue was fixed in paraformaldehyde, processed and embedded in paraffin, sectioned at 10 μm thickness, and stained with hematoxylin and eosin. A morphometric analysis was carried out using the automated software Adiposoft (54). Images for the analysis were acquired using a workstation comprising a microscope (Olympus BX-51; Olympus Corp., Tokyo, Japan) equipped with a CCD video camera (PixeLINK; Ottawa, ON, Canada). The whole system was controlled with the new CAST software package (Visiopharm Integrator System, version 3.2.7.0; Visiopharm, Denmark). Three high-resolution, randomly located images per section were acquired at 10× magnification. Cell area and diameter were determined using 100 adipocytes per section, three sections per animal, and five animals per group. The proportion of area covered with small adipocyte islets was determined in each section as well and presented in percentage.

### SDS Polyacrylamide Gel Electrophoresis and Western Blotting

The concentration of proteins in each sample was determined by the method of Lowry et al. (55). The samples were mixed 1:1 with 2× Laemmli's buffer and boiled for 5 min. Proteins (50 μg) were separated by electrophoresis through sodium dodecyl sulfate (SDS) polyacrylamide gels (7.5% or 12%) and transferred onto polyvinylidene fluoride (PVDF) membranes. The membranes were blocked with 3% bovine serum albumin (BSA) or 3% nonfat dry milk and incubated with appropriate primary antibody overnight at 4°C in order to detect NFκB (p65 subunit), pIRS1(Ser307), IRS1, Akt, pAkt (Ser473), PTP1B, F4/80, PPARγ, SOD1, SOD2, GRed, GPx, and CAT. Primary antibody was followed by HRP-conjugated appropriate secondary antibodies (1:30,000). Protein load correction in all the samples was conducted by probing membranes for β-actin (cytosols) and Lamin B (nucleosols) and respective secondary antibody. Immunopositive bands were visualized by the ECL reaction. A quantitative analysis of immunoreactive bands was performed using the ImageJ software.

### RNA Extraction and Reverse Transcription

Total RNA was extracted from thawed VAT (100–200 mg) using TRIzol^®^ Reagent following the protocol of the manufacturer. RNA was dissolved in 30 μl of RNase-DNase free water and an RNase inhibitor was added. Concentration and purity were tested spectrophotometrically (OD 260/280 > 1.8 was considered satisfactory). RNA integrity was confirmed by 1% agarose gel electrophoresis. Prior to cDNA synthesis, DNA contamination was removed by DNAse I treatment (Fermentas), according to the instructions of the manufacturer. cDNA was synthesized from 2 μg of RNA. Reverse transcription was performed in a 20-μl reaction with MultiScribe^TM^ Reverse Transcriptase in the presence of random primers using High-Capacity cDNA Reverse Transcription Kit. The reactions were carried out under RNase free conditions at 25°C for 10 min followed by 37°C for 2 h and final denaturation at 85°C for 5 min. The cDNA was stored at −80°C until further use.

### Real Time PCR

The expression of IL-1β, IL-6, and TNFα was analyzed by TaqMan qPCR using a QuantStudio3 sequence detection system. All the reactions were performed in 25 μl volume in triplicates and mean a Ct value for each triplicate was used for further analysis. Reaction mix consisted of 1 × TaqMan^®^ Universal PCR Master Mix with AmpErase UNG, 1 × TaqMan^®^ Gene Expression Assay, and a cDNA template (20 ng of RNA converted to cDNA). Thermal cycling conditions were: 2 min incubation at 50°C for UNG activation, 10 min at 95°C followed by 40 cycles of 95°C for 15 s and 60°C for 60 s. No template control was included for each target gene to detect possible reagent contamination. Relative quantification of gene expression was performed using comparative 2^−ΔΔCt^ method. HPRT1 was used as reference gene.

### Measurement of Xanthine Oxidase Activity

For the measurement of xanthine oxidase activity, the adipose tissue was homogenized in 100 mMTris buffer (pH 7.4) containing protease inhibitors and sonicated (3 × 15 s at 10 MHz on ice) prior to 20 min centrifugation at 20,000 × g (4 °C). Hepatic cell extracts were prepared as described previously (56). Xanthine oxidase activity was measured spectrophotometrically on the Synergy H1 Hybrid Multi-Mode Reader (BioTek Instruments, Winooski, VT, United States) by estimating the rate of oxidation of xanthine to uric acid at 295 nm (57). One unit of xanthine oxidase activity was defined as the amount of enzyme needed for production of one micromole of uric acid per minute at 37°C and pH 7.4. Enzyme activity is expressed as units (U) per mg of protein. The molar extinction coefficient of uric acid was ε_295_ = 12.5 mM^−1^ cm^−1^.

### Statistical Analysis

To compare differences between the experimental groups, Student's *t*-test (two-tailed) was performed. A probability level of <0.05 was considered to be statistically significant. Data are presented as mean ± SEM.

## Results

### Energy Intake, Adiposity, and Histological Analysis of VAT

**Figure 1:**
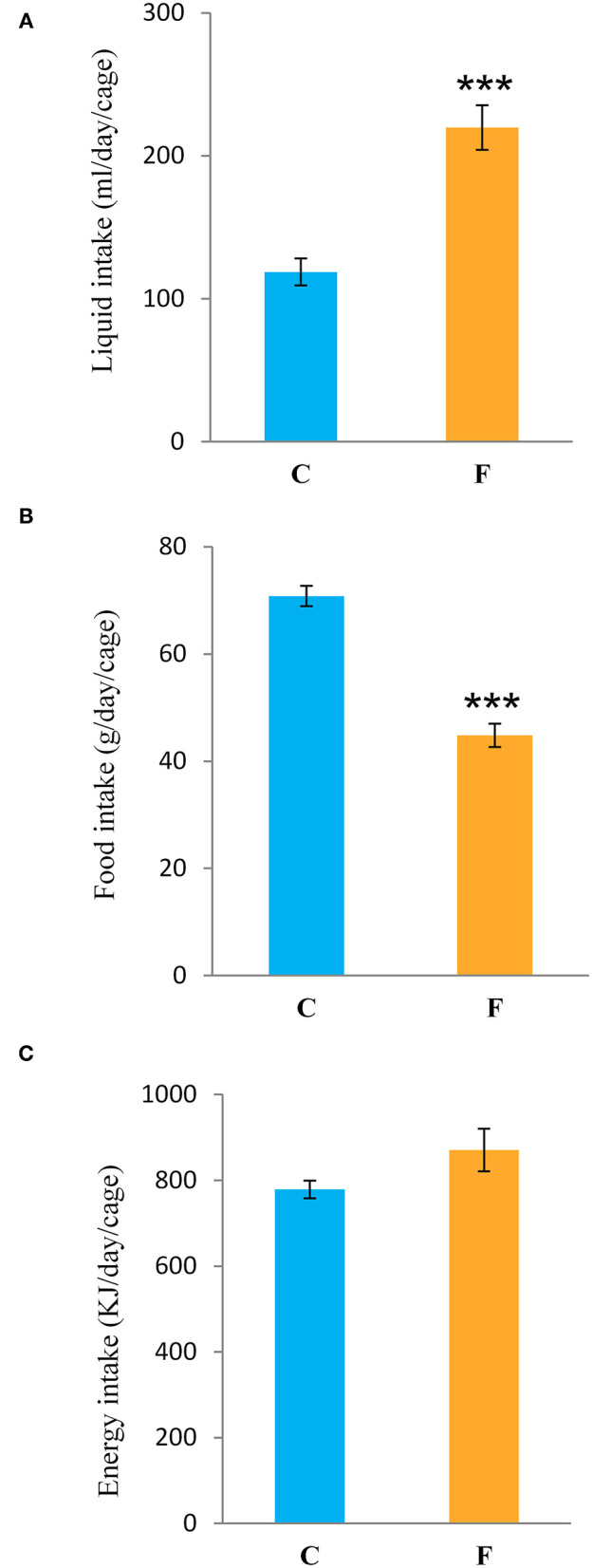
Liquid, food, and energy intake of female rats fed with 10% fructose diet for 9 weeks. **(A)** Liquid, **(B)** food, and **(C)** energy intake were measured per day and per cage. Values are expressed as mean ± SEM (*n* = 3 cages). Statistical significance of between-group differences (Student's *t*-test): ***P < 0.001, F vs. C. C, control group; F, fructose-fed group.

**Figure 2:**
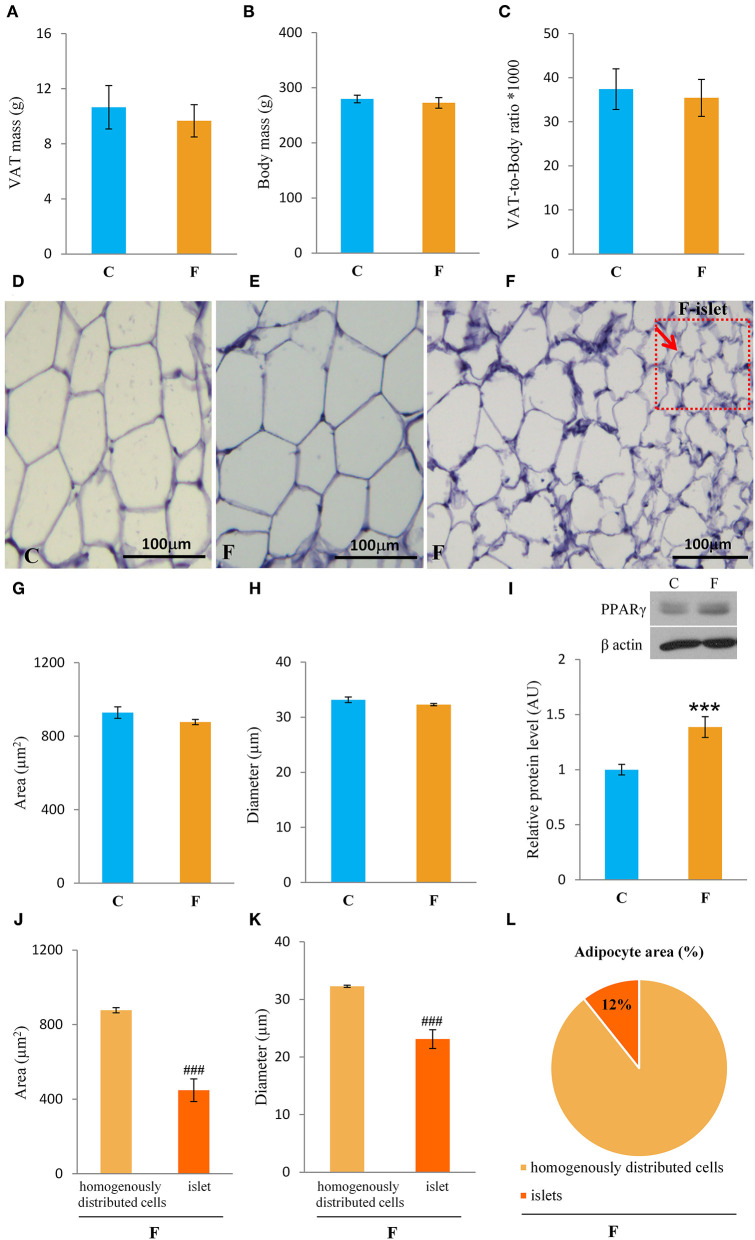
Characterization of visceral adipose tissue (VAT) from female rats fed with 10% fructose diet for 9 weeks. **(A)** VAT mass, **(B)** body mass, **(C)** VAT-to-body ratio, **(D)** representative histological VAT sections (magnification ×10) stained with hematoxylin and eosin of control, **(E)** fructose-fed female rats and **(F)** small adipocyte islets in the fructose-fed group, adipocyte **(G)** area and **(H)** diameter of control and fructose-fed groups, protein level of **(I)** PPARγ, adipocyte **(J)** area and **(K)** diameter from islets of the fructose-fed group compared with homogenously distributed cells in the same group and **(L)** the percentage of small adipocyte islets area in total adipocytes area. Values for VAT mass, body mass, and Western blot represent the mean ± SEM (*n* = 8 animals per group). For histology, three images were made per section, three sections per animal, and five animals per group. Statistical significance of between-group differences (Student's *t*-test): ***P < 0.001, F vs. C, ^###^*P* < 0.001, islets vs. homogenously distributed cells in the F group. C, control group; F, fructose-fed group.

Both female and male rats that consumed the fructose-enriched diet had higher liquid intake (Figures 1A, **4A**, *P* < 0.001) but lower solid food ingestion (Figures 1B, **4B**, *P* < 0.001) compared with the animals on standard diet. The energy intake of female rats on the fructose diet (Figure 1C) remained unchanged, while the male rats fed with fructose had increased energy intake (**Figure 4C**, *P* < 0.001). VAT mass, body mass, and VAT to body ratio were unaltered in both the female (Figures 2A–C) and male rats (**Figures 4D–F**). A histological analysis of VAT from the females reveled that adipocytes of the fructose-fed rats had unchanged diameter and area compared with the adipocytes of the rats on standard diet (Figures 2D,E,G,H). Nevertheless, VAT of female fructose-fed rats had islands of small adipocytes whose diameter and area were significantly smaller (*P* < 0.001) than the diameter and area of other cells in the VAT (Figures 2F,J,K). As shown in the Figures 2F,L, the percentage of these islets of small adipocytes is around 12%. The presence of small-sized adipocytes (around 20 μm in diameter) in the VAT of the fructose-fed female rats was parallel with the elevated level of PPARγ (Figure 2I), a transcriptional factor known as master regulator of adipogenesis. However, PPARγ protein level in the VAT of the male rats on fructose diet remained unchanged (Figure 4L).

### Systemic Insulin Sensitivity

**Figure 3:**
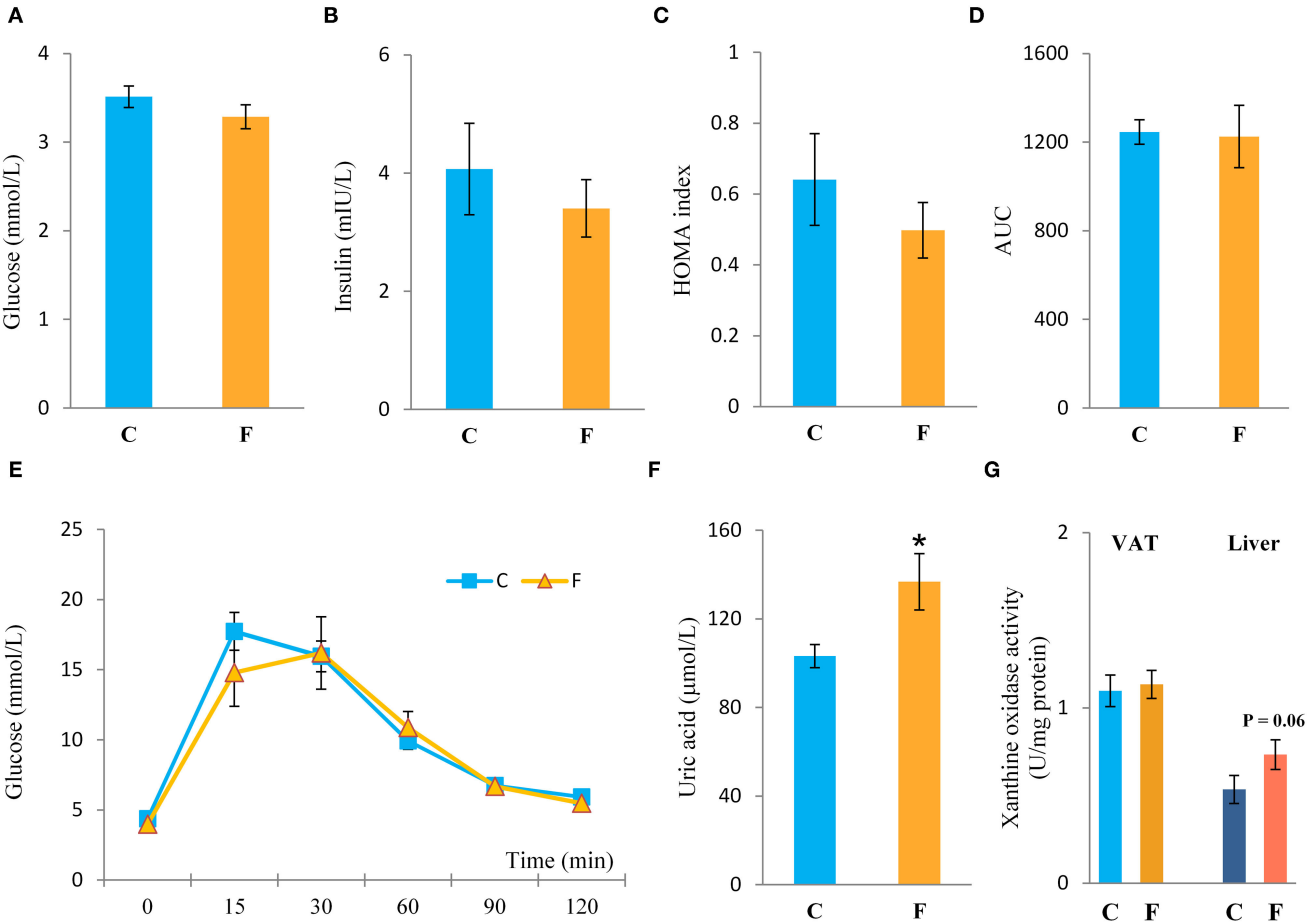
Systemic insulin sensitivity in female rats fed with 10% fructose diet for 9 weeks. **(A)** Blood glucose level, **(B)** plasma insulin level, **(C)** homeostasis model assessment (HOMA) index, **(D)** AUC values, **(E)** intraperitoneal glucose tolerance test (IPGTT), **(F)** plasma uric acid level, and **(G)** activity of xanthine oxidase in VAT and liver were determined in the control (C) and fructose-fed (F) female rats. The values represent the mean ± SEM (*n* = 8 animals per group). Statistical significance of between-group differences (Student's *t*-test): *P < 0.05, F vs. C.

**Figure 4:**
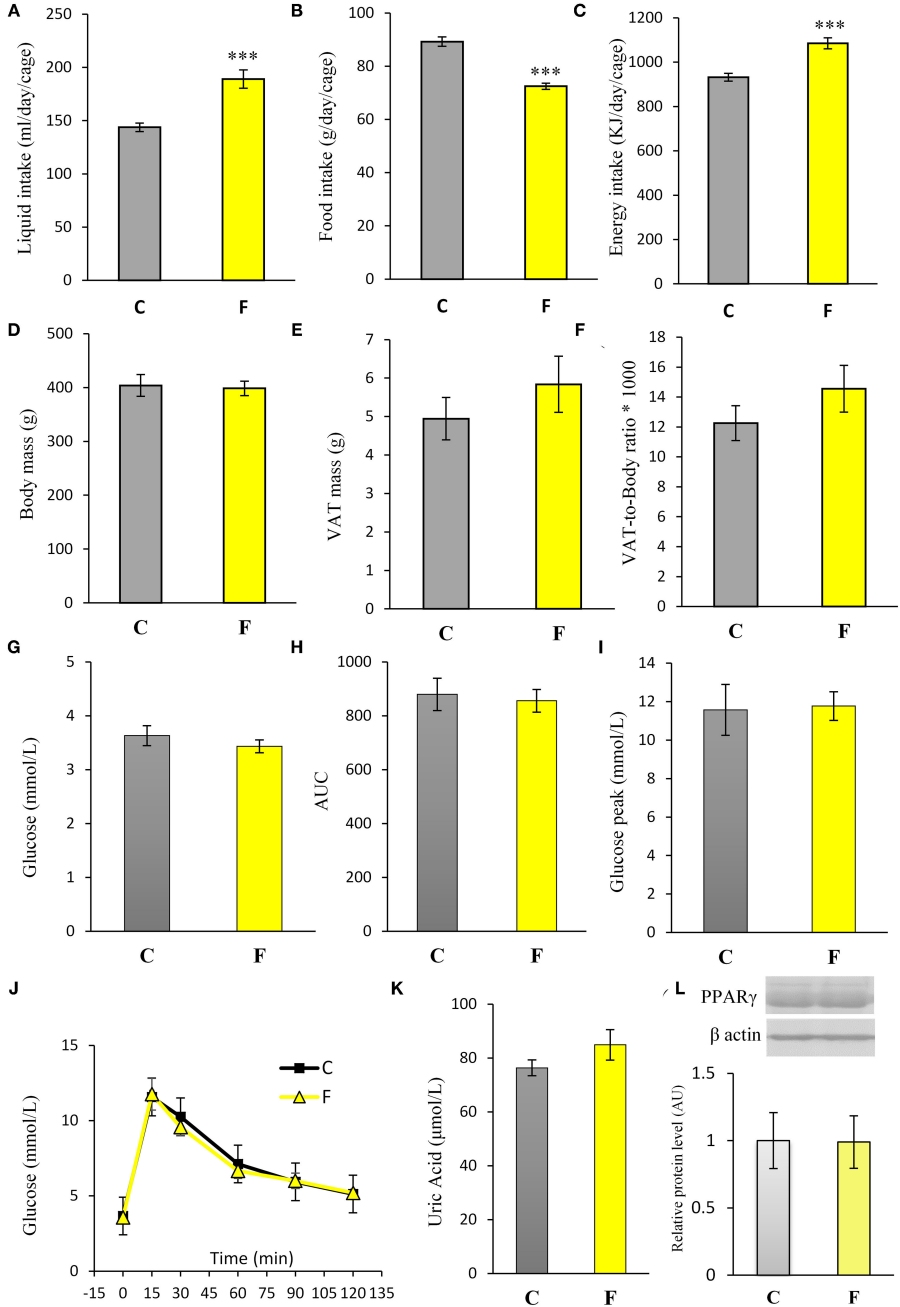
Physiological and biochemical parameters of male rats fed with 10% fructose diet for 9 weeks. **(A)** Liquid, **(B)** food, and **(C)** energy intake were measured per day and per cage. Values are expressed as mean ± SEM (*n* = 3 cages). **(D)** Body mass, **(E)** VAT mass, and **(F)** VAT-to-body ratio; **(G)** blood glucose level, **(H)** AUC values, **(I)** glucose peak, **(J)** IPGTT, **(K)** plasma uric acid level and **(L)** PPARγ protein level were determined in the control (C) and fructose-fed (F) male rats. The values represent the mean ± SEM (*n* = 8 animals per group). Statistical significance of between-group differences (Student's *t*-test): ***P < 0.001, F vs. C.

Both the female and male fructose-fed rats had unchanged glucose levels (Figures 3A, 4G). Insulin (Figure 3B), as well as the calculated HOMA index (Figure 3C), was not changed by the fructose-enriched diet in females. Response to intraperitoneal glucose application was not changed by fructose consumption in both sexes, since IPGTT (Figures 3E, 4J) and calculated AUC (Figures 3D, 4H) values showed no statistical difference between fructose and the control group of the animals. Also, glucose peak in the males remained unaltered by fructose feeding (Figure 4I). On the other hand, level of uric acid in the circulation of female rats was increased after long term fructose diet (Figure 3F, *P* < 0.05), while in male rats the level of uric acid was unchanged after the same diet (Figure 4K). The activity of xanthine oxidase exhibited an increasing trend in the liver of the fructose-fed female rats in comparison with the control group (*P* = 0.06), while it remained unaltered in the VAT after fructose diet (Figure 3G).

### VAT Insulin Signaling

**Figure 5:**
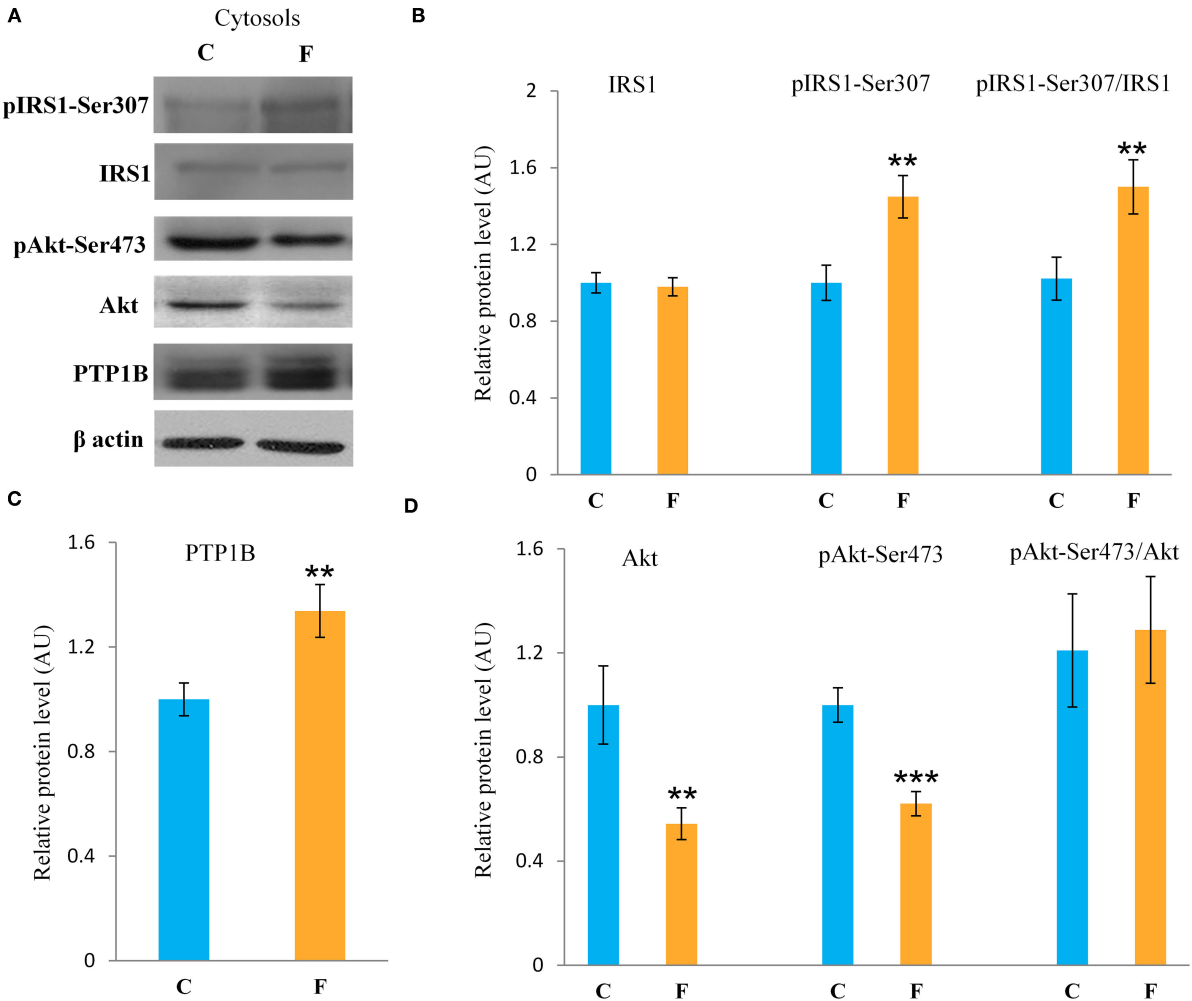
Insulin signaling in the VAT of female rats fed with 10% fructose diet for 9 weeks. Representative **(A)** Western blots, protein levels of IRS1, pIRS1-Ser307 and its ratio to total IRS1 **(B)**, protein level of **(C)** PTP1B, protein levels of Akt, pAKT-Ser473 and its ratio to total Akt **(D)**. All protein levels were measured by Western blot in the cytosols of the VAT from control (C) and fructose-fed (F) female rats, normalized to β actin and expressed in arbitrary units (AUs). The values represent the mean ± SEM (*n* = 8 animals per group). Statistical significance of between-group differences (Student's *t*-test): **P < 0.01 and ***P < 0.001, F vs. C.

The VAT insulin signaling pathway was assessed at the protein level of total IRS1, its inhibitory phosphorylation on Ser307, protein level of total Akt, and its stimulatory phosphorylation on Ser473. The protein level of PTP1B, main inhibitor of the insulin signaling pathway, was determined as well. Western blot results for females (Figure 5A) showed that the fructose-fed animals had increased protein level of pIRS1-Ser^307^ and its ratio to total IRS1 (*P* < 0.01, Figure 5B), and that the level of Akt (*P* < 0.01) and pAkt-Ser^473^ (*P* < 0.001) were drastically decreased (Figure 5D). Fructose consumption also led to increase at the PTP1B protein level in the VAT of the female rats on fructose diet in comparison with the animals on standard diet (*P* < 0.01, Figure 5C). However, among the examined parameters of VAT insulin resistance in males (**Figures 7A–D**), only the ratio of pAkt-Ser^473^ to total Akt was decreased after fructose diet.

### VAT Inflammation

**Figure 6:**
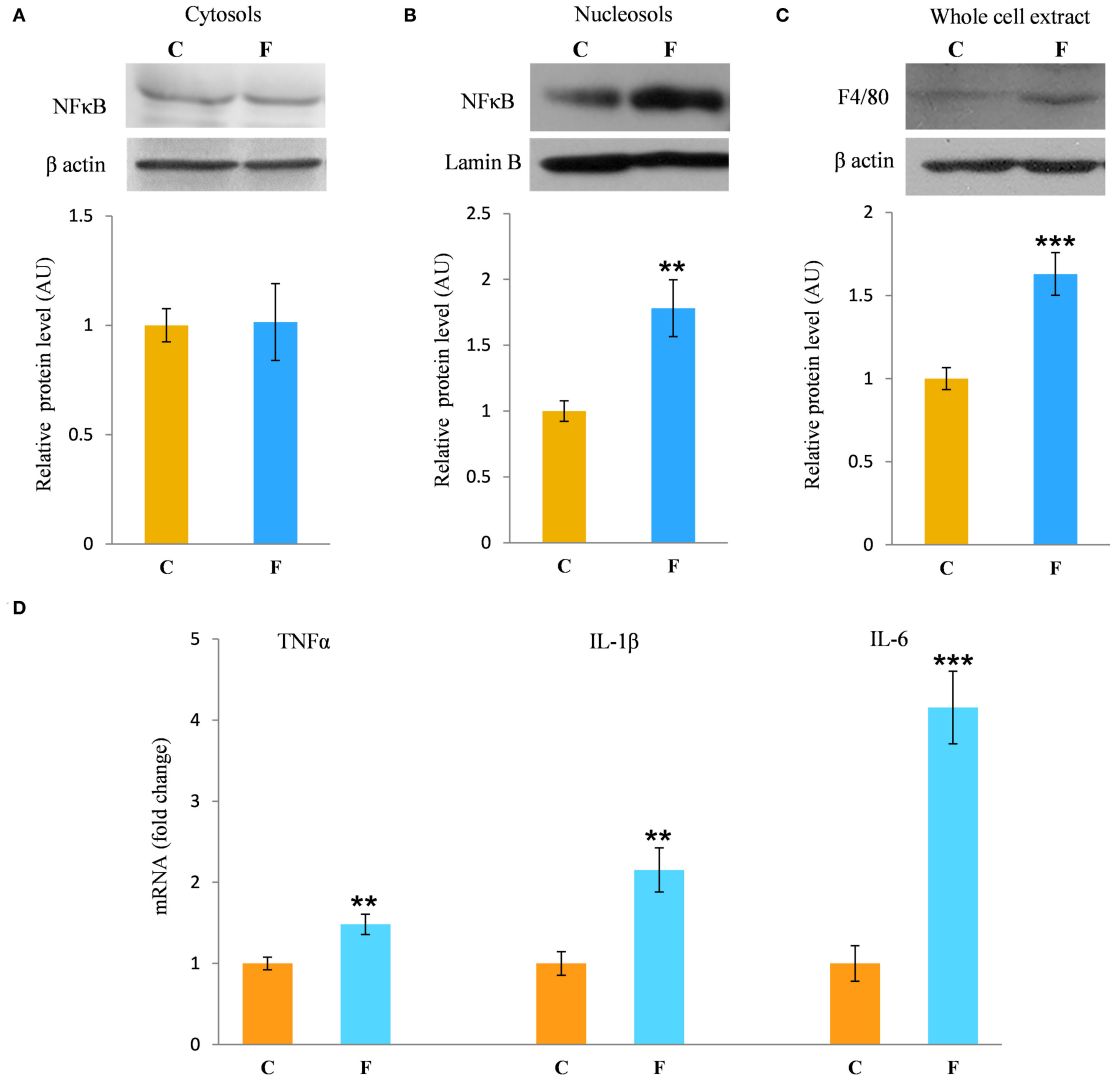
Inflammation in the VAT of female rats fed with 10% fructose diet for 9 weeks. Protein level of NFκB in **(A)** cytosols and **(B)** nucleosols, and protein level of **(C)** F4/80 in whole cell extracts were measured by Western blot in the VAT from control (C) and fructose-fed (F) rats, normalized to β actin and expressed in AUs. The values represent the means ± SEM. Statistical significance of the difference between experimental groups (Student's *t*-test): **P < 0.01 and ***P < 0.001, F vs. C. The level of TNFα, IL-1β, and IL-6 mRNAs relative to HPRT mRNA were determined by TaqMan real-time PCR in the VAT **(D)**. The values represent the mean ± SEM (*n* = 8 animals per group). All measurements were done in triplicate. Statistical significance of the difference between experimental groups (Student's *t*-test): **P < 0.01 and ***P < 0.001, F vs. C.

**Figure 7:**
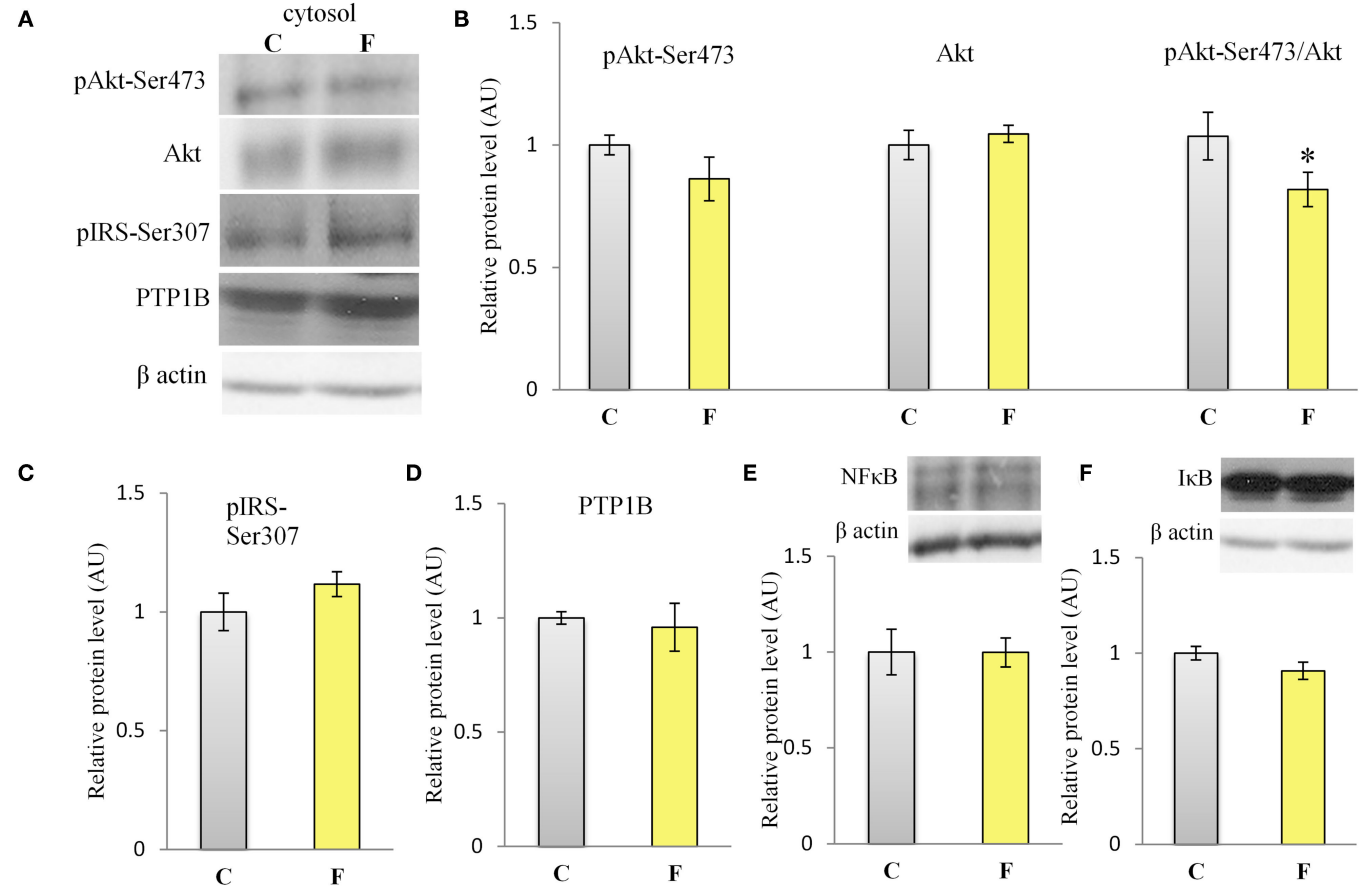
Insulin signaling and inflammation in the VAT of male rats fed with 10% fructose diet for 9 weeks. **(A)** Representative Western blots **(A)** and relative quantification for protein levels of Akt, pAkt-Ser473 and its ratio to total **(B)** Akt, **(C)** pIRS1-Ser307, **(D)** PTP1B, **(E)** NFκB, and **(F)** IκB. All protein levels were measured by Western blot in the cytosols of the VAT from control (C) and fructose-fed (F) female rats, normalized to β actin and expressed in arbitrary AUs. The values represent the mean ± SEM (*n* = 8 animals per group). Statistical significance of between-group differences (Student's *t*-test): *P < 0.05, F vs. C.

The inflammatory status of VAT after long-term fructose consumption was determined at the level of cellular distribution of NFκB, one of the main proinflammatory transcriptional regulators that have been correlated with diet-induced disturbances. The potential change in the transcription of the genes for proinflammatory cytokines TNFα, IL1-β, and IL-6, regulated by NFκB, was assessed by measuring their mRNA level in the VAT. The protein level of macrophage marker F4/80 was determined as well. The results for the female rats showed unchanged NFκB protein level in the VAT cytosols (Figure 6A) after fructose consumption, while fructose led to increase in the NFκB protein level of the nucleosols (*P* < 0.01, Figure 6B). This change indicates nuclear translocation of NFκB. In accordance with this, the fructose-fed female rats had increased level of TNFα (*P* < 0.01), IL-1β (*P* < 0.01), and IL-6 (*P* < 0.001) mRNA (Figure 6D), as determined by RT-PCR. The protein level of F4/80 was increased in the VAT of the fructose fed female rats as well (*P* < 0.001, Figure 6C). In contrast, both NFκB and IκB protein levels remained unchanged in the VAT of the male rats (Figures 7E,F).

### VAT Antioxidative Defense System

**Figure 8:**
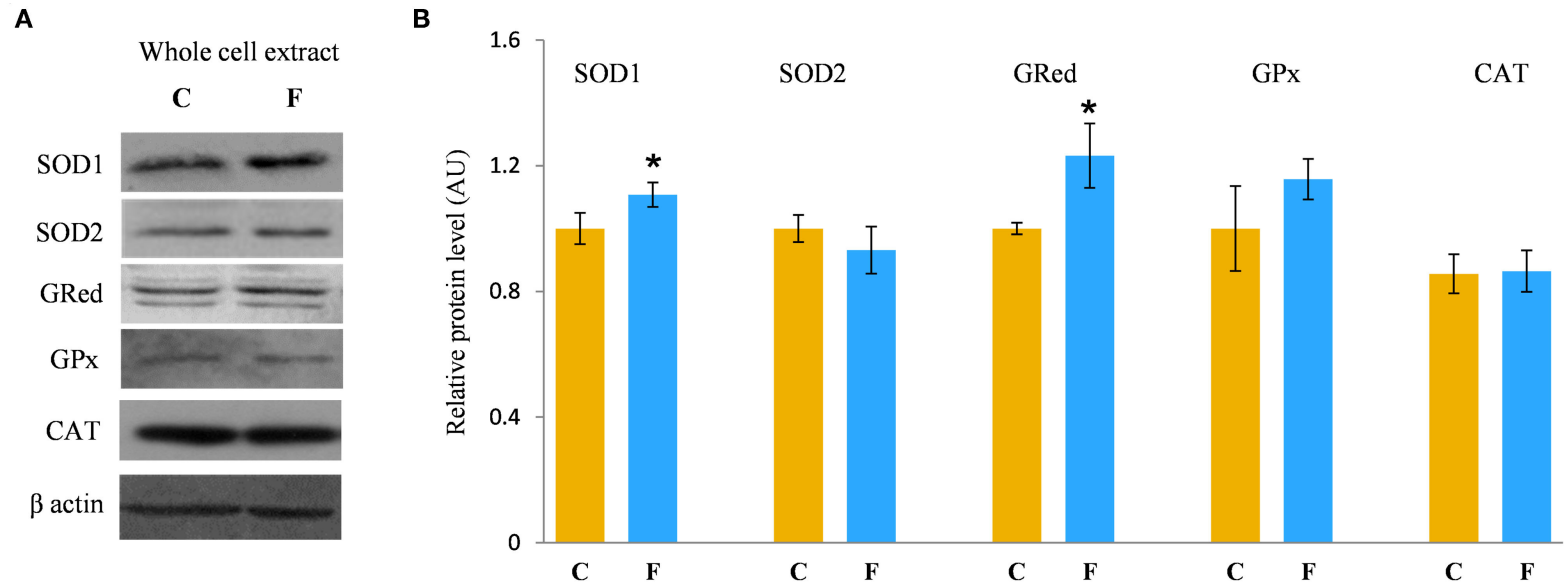
Antioxidative enzymes in the VAT of female rats fed with 10% fructose diet for 9 weeks. Representative Western blots **(A)** of the protein levels of SOD1, SOD2, GRed, GPx, and CAT **(B)** were measured by Western blot in the whole cell extracts of the VAT from control (C) and fructose-fed (F) rats, normalized to β actin and expressed in AUs. The values represent the means ± SEM (*n* = 8 animals per group). Statistical significance of the difference between experimental groups (Student's *t*-test): *P < 0.05, F vs. C.

In order to evaluate the antioxidative defense system in the VAT, we determined the protein levels of SOD1, SOD2, GRed, GPx, and CAT, main enzymes involved in the protection of cells from ROS. The results showed increased protein levels of SOD1 (*P* < 0.05) and GRed (*P* < 0.05) in the VAT of the fructose-fed female rats in comparison with the control group. There was no difference in the protein levels of SOD2, GPx, and CAT after fructose-enriched diet (Figures 8A,B).

## Discussion

The results of our study show that liquid the fructose-enriched diet induces inflammation and insulin resistance in the VAT of the adult female rats, and that the same diet does not elicit such changes in the VAT of the male rats. Although, in the females the fructose diet initiated the development of a distinctive population of small adipocytes, it did not affect VAT mass. The presence of VAT insulin resistance and newly formed adipocytes in the females, although without obesity, suggests that adipose tissue dysfunction, rather than its simple enlargement, plays a role in the development and progression of insulin resistance-related metabolic disorders.

Fructose is a highly palatable sugar, and rats exposed to a fructose solution tend to increase liquid and, hence, energy intake. Indeed, in our study, both the female and male rats that consumed the fructose solution had marked increase in liquid intake in comparison with the control animals. Nevertheless, the fructose-fed female rats consumed the same energy as the control rats as a consequence of the lower solid food intake in these animals, which probably served as a compensation mechanism for the calories that originated from fructose. Although there are studies (including our own) showing full compensation and, thus, higher energy intake with fructose diet (44, 45, 58, 59), we propose that the similar energy intake observed between the experimental groups in females possibly originates from the unchanged expression of AgRP, NPY, POMC, and CART, previously shown in the hypothalamus of the same animals (60). Although fructose diet has been associated with increased VAT mass and obesity, in our study, fructose overconsumption was not followed by increased body mass and VAT mass or by higher VAT-to-body ratio in females, which goes in line with unchanged daily energy intake. It is noteworthy that other studies on female rats on the same dietary regime reported increased VAT mass and adipocyte hypertrophy. The discrepancies between the results might stem from differences in treatment duration (ranging from 9 weeks to 7 months), age of the animals at the beginning of the treatment (3–8 weeks), and strain of the experimental animals (61). Additionally, the limitation of this study is that the results were obtained at a single time point, after 9 weeks of diet, so it could be speculated that prolonged treatment could lead to more pronounced effects. Interestingly, our previous study on young female rats on fructose diet showed an increase in energy intake and development of obesity, indicating that young, preadult female rats may be more prone to the obesogenic effects of fructose and that sex hormones may have a protective role (49). In line with this is the study of Galipeau et al. (62) showing that ovariectomy makes female rats more sensitive to metabolic effects of fructose-feeding and development of hypertension and hyperinsulinemia. In contrast to the females in this study, the male rats subjected to the same diet had increased energy intake, while their body and VAT mass also remained unchanged. Increased energy intake in males most likely originates from calories ingested as solid food, which goes in line with studies on human subjects showing that men consume more daily energy than women (63).

In accordance with unchanged VAT mass in the females, adipocyte area and diameter did not change between the fructose-fed and control animals. However, the formation of the islets of a new, distinctive population of smaller adipocytes (around 20 μm in diameter) was observed in the VAT of the fructose-fed rats. Indeed, it has been shown by several authors that fructose-feeding has a potential to directly induce differentiation of pre-adipocytes in the cell culture. Namely, Zubiria et al. (64) observed that after exposure to fructose (but not glucose), adipocyte precursor cells are more capable of generating new adipocytes and that 10% fructose solution initiated the development of small adipocyte islets in male Sprague-Dawley rats. The presence of small-sized adipocytes in the VAT of the fructose-fed female rats in our study was parallel with the elevated level of PPARγ, a transcriptional factor known as master regulator of adipogenesis (65), which further corroborates the assumption that these are new adipocytes, together with our previous finding of adipogenesis in the VAT of young male rats fed with 60% fructose diet (66). However, in this study, PPARγ remained unchanged in the VAT of the adult males fed with 10% fructose solution, which can imply the absence of adipogenesis. Although women do not consume more energy compared with men, they maintain a greater percentage of body fat mass from puberty to menopause (63). It seems that this is due to increased fat storage capability in females. Indeed, previous studies have demonstrated that efficient fat storage in women was mediated through reduced postprandial fatty acid oxidation most likely because of the influence of estrogens on hepatic fat processing (67). In line with this, the hepatic ApoE gene was shown to be expressed differently in male and female rats (68).

The presence of small adipocytes has been previously related to the improvement of tissue and systemic insulin sensitivity. Although in our study systemic insulin sensitivity after 9 weeks of fructose consumption was unchanged, judged by unaltered blood glucose and insulin levels, as well as the HOMA index and IPGTT, our female rats on fructose diet had disturbed VAT insulin signaling, as indicated by the decreased protein level of total Akt and its activating Ser473 phosphorylation, together with the increased level of inhibitory phosphorylation of IRS1 on Ser 307 and increased level of PTP1B. Similar to the females, the male rats on the same fructose diet also had unchanged glucose levels and IPGTT parameters, while among the markers of insulin resistance in the VAT, only pAkt to total Akt ratio was decreased. Nevertheless, Kubacka et al. (69) recently showed increased pIRS1-Ser^307^ and its ratio to total IRS1 in the adipose tissue of 20% fructose-fed male rats for 18 weeks. Although insulin signaling impairment in the VAT has often been correlated with enlarged hypertrophic adipocytes (70), there are studies reporting no differences in the diameter between large adipocytes from insulin-sensitive and insulin-resistant BMI-matched subjects, who actually show an excess of small “immature” adipocytes compared with larger cells (71). It was even postulated that insulin resistance could be more closely related to the presence of small adipocytes rather than the large, hypertrophic ones (72). These smaller cells possibly fail to mature into larger cells, thus limiting fat storage capacity in the adipose tissue and redirecting excess fat redistribution to ectopic sites. However, normal-weight individuals may also be insulin resistant, suggesting that overall adiposity is not the sole determinant of insulin resistance (73). Additionally, other authors reported increased presence of small adipocytes in diabetic patients in comparison with control subjects with matched BMI (74). Interestingly, Liu et al. (72) showed that small adipocytes from obese Zucker rats have increased transcription level of IL-6 and proinflammatory activity in comparison with total adipocyte population present. It seems that adipose tissue dysfunction, rather than its simple enlargement, significantly contributes to the onset and development of metabolic disorders (75).

There has been a growing interest in VAT inflammation in obese and non-obese subjects, since the presence of low-grade chronic inflammation was described in patients suffering from different metabolic disorders (76). The results presented in this study clearly show that fructose consumption stimulated the development of inflammation in the VAT of female rats. Namely, nuclear accumulation of NFκB was observed in animals on fructose diet, indicating the activation of this pro-inflammatory transcription regulator. This was further supported by the elevated expression of NFκB-regulated pro-inflammatory cytokines, TNFα, IL-1β, and IL-6, and increased protein level of macrophage marker F4/80 in the VAT of the fructose-fed females. This result was not surprising, since there are literature data associating fructose consumption with the development of chronic, low-grade inflammation in different tissues, although majority of the studies were conducted on males. Fructose diet was shown to activate NFκB in the heart of male rats (77), increase IL-1β and TNFα level in the liver (78) and blood plasma (79–81), and elevate the production of inflammation markers, such as TNFα and IL-6 in the VAT (82, 83). However, our males fed with 10% fructose solution for 9 weeks did not show signs of VAT inflammation, at least at the level of NFκB activation. One of the rare studies conducted on females reported increased TNFα, IL-1β, and IL-6 level in the VAT after 24 weeks of 10% fructose diet (14). This is similar to our results, although we showed the presence of VAT inflammation much earlier, after 9 weeks of fructose diet. One of the proposed factors with which fructose diet could elicit inflammation is the uric acid generated during fructose metabolism (84). Namely, fructose phosphorylation by ketohexokinase leads to rapid and transient depletion of ATP, consequent increase in purine degradation with xanthine oxidase, and generation of uric acid (85). Both human and animal studies have shown fructose-induced hyperuricemia (86), which could, thus, be related to enhanced xanthine oxidase activity in the liver and small intestine, reduced renal uric acid excretion, and/or increased uric acid reabsorption (87). Adipose tissue was also shown to produce and secrete uric acid in both *in vitro* and *in vivo* studies (88). Furthermore, when adipose tissue was treated with fructose, rise in uric acid was registered together with drastic increase in adipogenesis (89). However, unaltered xanthine oxidase activity in the VAT and the trend of its increased activity in the liver, observed in this study, suggest that the increase in plasma uric acid observed in the females could rather be related to the liver and not to the adipose tissue itself. Nevertheless, it has been shown that the incubation of adipocytes with uric acid induced PPARγ expression, increased adipogenesis, and raised the expression of NADPH oxidase and superoxide anion levels (89), which goes in line with the increased SOD1 level observed in this study. Furthermore, uric acid was found to induce adipose tissue inflammation, as evidenced by increased level of inflammatory cytokines in cultured adipocytes (90). Considering this, the elevated plasma uric acid observed herein could contribute to VAT inflammation and, additionally, uric acid could also be the possible link through which fructose is initiating adipogenesis in female rats. This could be example of the systemic effects of fructose through liver-VAT crosstalk and the way in which liver can indirectly contribute to VAT metabolic dysfunction. Our recently published results on the liver of the same fructose-fed female rats (91) showed changes only in AMPK activation, while effects on hepatic *de novo* lipogenesis, lipid excretion, and inflammation were absent. Having this in mind, together with the observed insulin signaling impairment and induced gene expression of proinflammatory cytokines in the VAT shown herein, it could be proposed that VAT dysfunction could be one of the earliest metabolic disturbances, and that intensive fructose metabolism in the liver can boost this process through uric acid production. In contrast to the females, the absence of VAT inflammation in the males goes in line with the unchanged levels of plasma uric acid, which could suggest that sex-related metabolic differences in response to fructose diet could be, at least partly, mediated by uric acid metabolism. Indeed, a recent study on humans showed that increased uric acid in serum is related to the risk of metabolic syndrome in females but not in males (92). Sex differences in the level of plasma uric acid, observed herein, could originate from differences in hepatic fructose metabolism, particularly the expression of the enzyme ketohexokinase. Namely, Vilà et al. (93) reported that fructose-related increase in hepatic ketohexokinase was much higher in females than in males and observed marked increase of the AMP/ATP ratio and raised AMPK activity in the liver of female but not of male fructose-fed rats.

The rise of chronic inflammation and development of obesity-related insulin resistance has been firmly connected with the infiltration of macrophages in the adipose tissue (22). Although this infiltration was usually associated with the expansion of VAT, it has been recently shown that macrophage infiltration can precede mass gain and adipocyte hypertrophy (94). This goes in line with our results on females showing increased level of the F4/80 macrophage marker, even though the VAT was not enlarged, but smaller adipocytes were present. It has been pointed out that the rise of VAT inflammation could have a regulatory role in the manner of adipose tissue remodeling and induction of catabolic processes in the conditions of chronic metabolic overload (95), such as the long-term fructose-enriched diet applied in our study. Others have reported that fructose can stimulate the transcription of inflammatory markers in vascular cells in the first hour of application (96), and that high carbohydrate diet can cause rise of adipose tissue inflammatory markers as fast as 1–3 days, persisting for 12 weeks (97). Furthermore, if adipose tissue inflammation is prolonged and not adequately resolved, it could cause insulin signaling impairment and related metabolic disturbances (98, 99). Since, in our study, inflammation was detectable after 9 weeks of fructose consumption, it could be assumed that it was probably not resolved fast enough and, thus, contributed to the observed insulin signaling impairment in the VAT of female rats.

The mechanisms behind inflammation-related insulin signaling impairment include TNFα, which can the promote inhibitory phosphorylation of IRS1 on Ser 307 (100, 101) and whose concentration has been correlated with adipose tissue IRS1 inactivation in obese patients (102). Furthermore, TNFα has the ability to activate NFκB and, therefore, promote its own transcription and expression of more proinflammatory cytokines, forming a vicious cycle. Activated NFκB was also shown to enhance the expression of PTP1B, a main inhibitor of the insulin signaling pathway (103). Similarly, IL-1β was shown to reduce IRS1 expression at both transcriptional and posttranscriptional levels (104), and the lack of IL-1β receptor improves glucose homeostasis and protects mice from developing adipose tissue inflammation after high-fat diet (105). IL-6 was associated with the development of insulin resistance as well, and visceral adipose tissue is considered an important source of its production (106). Studies also reported that the inhibition of TNFα and IL-6 prevented the development of insulin resistance in obese animals (31, 107). Macrophage infiltration and chronic inflammation are tightly correlated with the development of obesity-related insulin resistance as well (94). Furthermore, the selective depletion of macrophages from visceral adipose tissue protects mice on high-fat diet from the development of glucose intolerance and insulin resistance (108). This, together with the potential of fructose to change the endocrine function of adipocytes to proinflammatory state, can lead to chronic inflammation and development of VAT insulin resistance. The absence of inflammation in male rats could be related to less pronounced derangement in VAT insulin signaling in males.

It has been pointed out that numerous metabolic disturbances, such as insulin resistance, could be a consequence of redox imbalance originating from nutritional excess and oxidative stress (109). Fructose-enriched diet was shown to induce oxidative stress (40) and only 1 week of fructose consumption can increase ROS level in the aorta, heart, and circulation (110). The rise of ROS was also observed in the adipose tissue after 16 weeks of 35% fructose consumption in male rats (111). Several studies have shown that ROS production can stimulate signaling pathways that regulate cell proliferation and differentiation (112, 113), and ROS was found to be increased during adipogenesis *in vitro*, while the inhibition of ROS decreased differentiation to mature adipocytes (114). In addition, fructose can disrupt antioxidative defense by lowering the activity and/or expression of antioxidative enzymes in adipose tissue and other organs in female (115) and male (80) rats. We have previously reported fructose-induced visceral adiposity and reduced protein level of antioxidant enzymes in the VAT of young female rats (49). Current results, however, show the absence of adiposity and increased levels of SOD1 and GRed in the VAT of adult fructose-fed females, which most likely serve to prevent intracellular ROS accumulation and oxidative damage of macromolecules. It is possible that prolonged treatment might ultimately overcome the antioxidative defense to induce oxidative stress and contribute to further propagation of fructose-related metabolic disturbances.

Many *in vivo* studies investigating fructose effects on adipose tissue inflammation and insulin resistance have reported obesity and increase in adipose tissue mass but usually with increased caloric intake from fructose diet. Therefore, it was hard to distinguish the effects of fructose *per se* from the effects of energy overload. This study, however, clearly shows that fructose-rich diet, rather than caloric excess, is responsible for VAT inflammation and insulin resistance in female rats, since their total energy intake was unchanged. Furthermore, fructose intake initiated the proliferation of small-sized adipocytes, although obesity and adiposity had not developed yet. This suggests that VAT inflammation and consequential disturbance of VAT insulin signaling could be critical events, which can start even before measurable increase of VAT mass, making it a silent risk factor for the development of type 2 diabetes. However, fructose-feeding had more pronounced effects on VAT inflammation and insulin signaling in female than on male rats, and the observed distinction could possibly originate from sex-related differences in uric acid metabolism. In conclusion, our results suggest that adipose tissue dysfunction could be one of the earliest metabolic changes that precedes the development of obesity and associated metabolic disorders, and that these processes could be gender-dependent.

## Data Availability Statement

The original contributions presented in the study are included in the article/Supplementary Material, further inquiries can be directed to the corresponding author.

## Ethics Statement

The animal study was reviewed and approved by Ethical Committee for the Use of Laboratory Animals of the Institute for Biological Research “Siniša Stanković”, University of Belgrade (Permit No. 02-11/14 obtained on 13.11.2014).

## Author Contributions

SK, BB, and LG contributed on data acquisition. JB and DVM analyzed the data. IE contributed on data interpretation. SK and AD designed the study and wrote the article. All the authors participated in the critical review of the manuscript and approved its final version.

## Funding

This study was supported by the Ministry of Education, Science and Technological Development of the Republic of Serbia (451-03-9/2021–14/200007) and Swiss National Science Foundation, Grant SCOPES JRP IZ73Z0_152331.

## Conflict of Interest

The authors declare that the research was conducted in the absence of any commercial or financial relationships that could be construed as a potential conflict of interest.

## Publisher's Note

All claims expressed in this article are solely those of the authors and do not necessarily represent those of their affiliated organizations, or those of the publisher, the editors and the reviewers. Any product that may be evaluated in this article, or claim that may be made by its manufacturer, is not guaranteed or endorsed by the publisher.
