# Supplementary material for: Fructose Induces Visceral Adipose Tissue Inflammation and Insulin Resistance Even Without Development of Obesity in Adult Female but Not in Male Rats
Source: Front Nutr. 2021 Nov 11;8:749328. doi: 10.3389/fnut.2021.749328 (PMC8632624; doi:10.3389/fnut.2021.749328)
Supplement: Supplementary file 1 [file Data_Sheet_1.pdf]

ECL Storm® blot imaging system (Amersham Biosciences-GE Healthcare, Chicago, Illinois) or X-ray films (Kodak, Rochester, NY, USA)

1,5 – C
2,6 – F
3,7, 4, 8 – experimental groups not included in this manuscript

β-actin (IRS1, Akt)	1 2 3 4 5 6 7 8 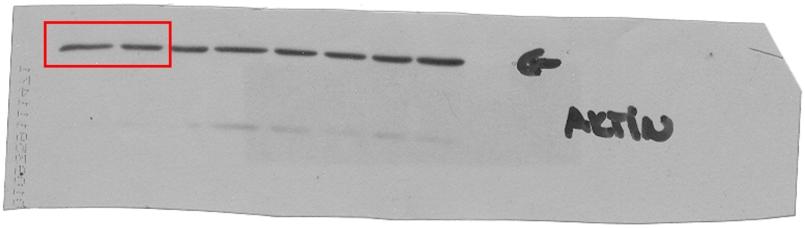
β-actin (pAkt-Ser473)	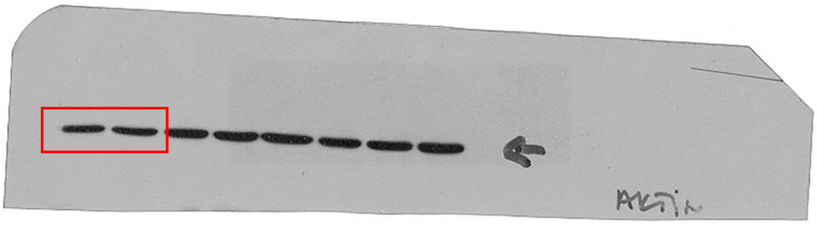
β-actin (pIRS1-Ser307, PTP1B)	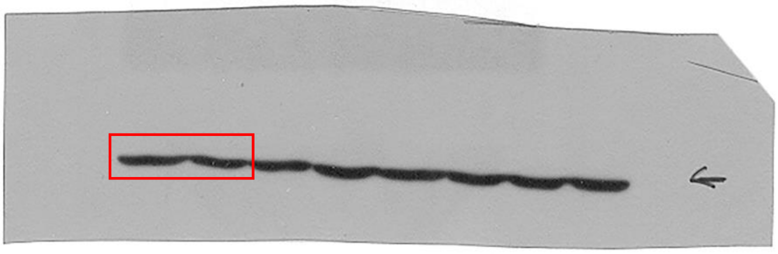
β-actin (F4/80)	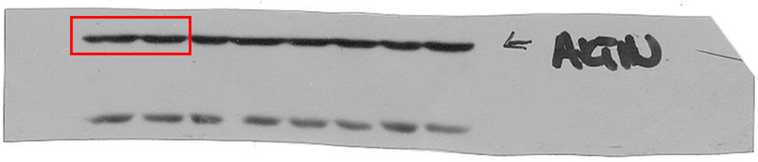
β-actin (NFκB cytosols)	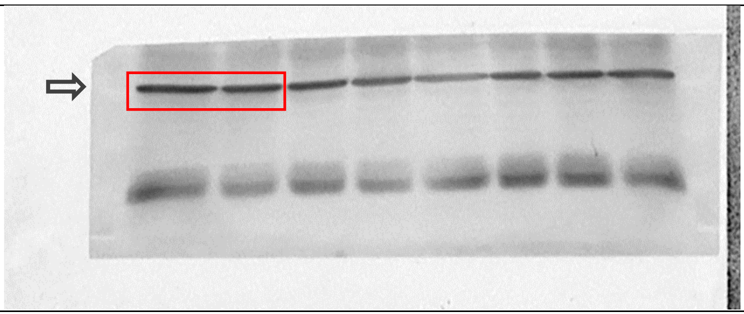

Lamin B (NFκB nucleosols)	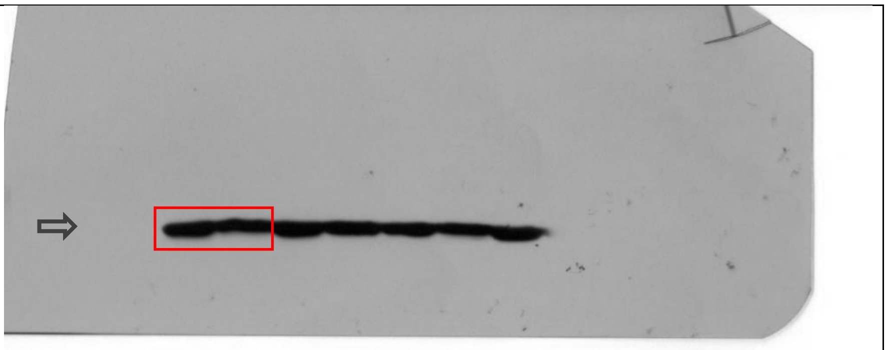
β-actin (GRed, GPx)	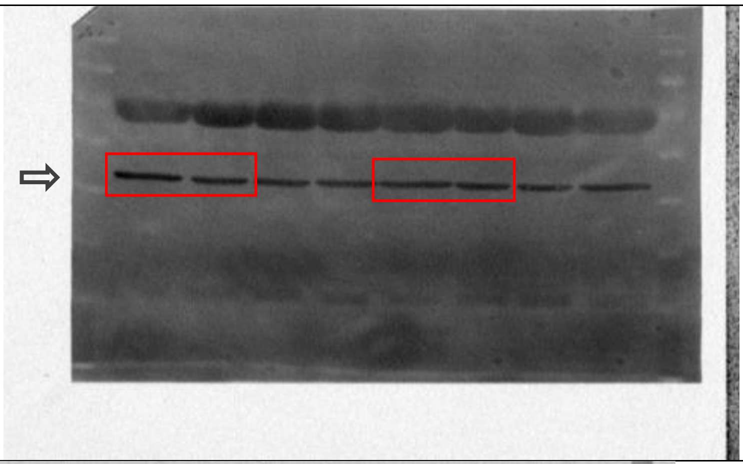
β-actin (SOD2)	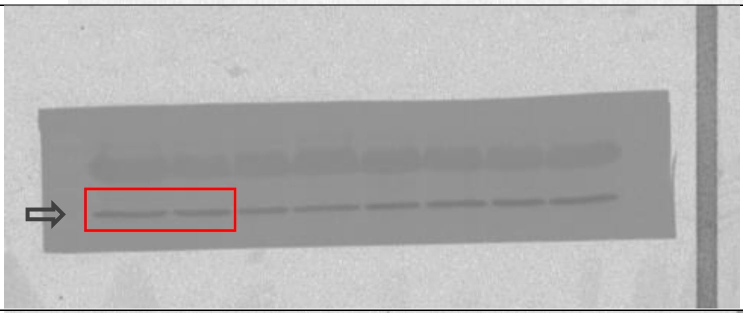
β-actin (SOD1)	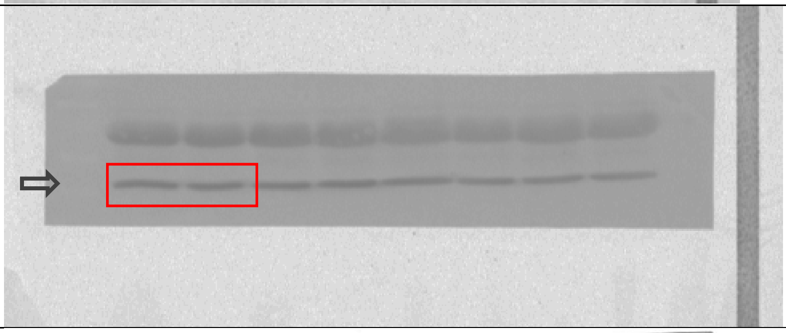
β-actin (CAT)	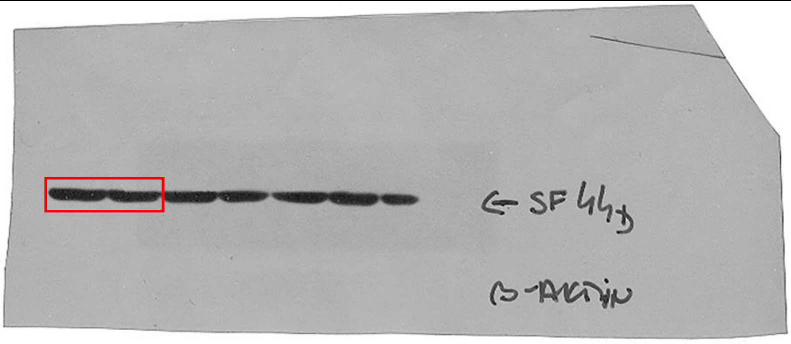

Akt (62kDa)	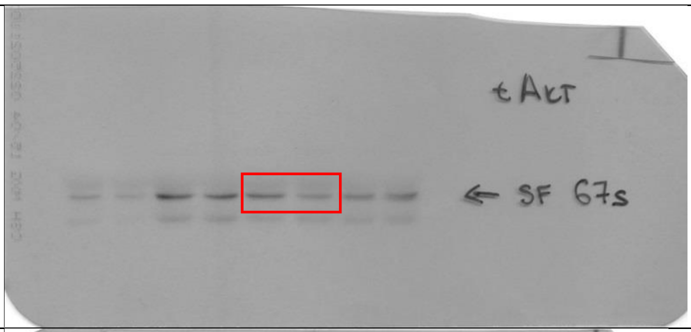
pAkt-Ser473 (62kDa)	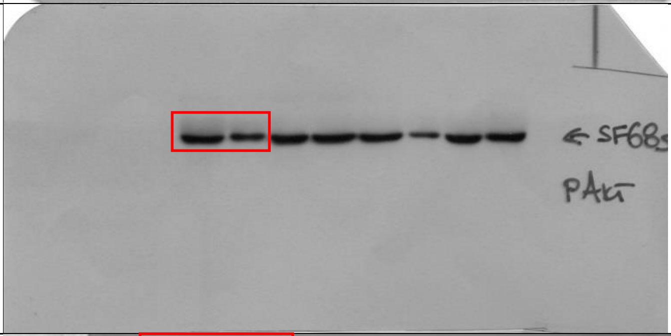
IRS1 (180kDa)	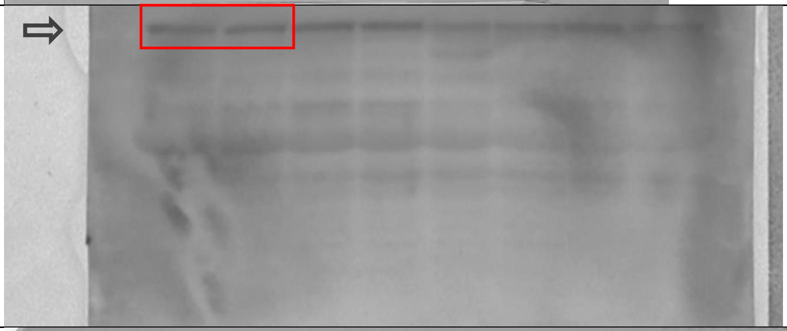
pIRS1-Ser307 (180kDa)	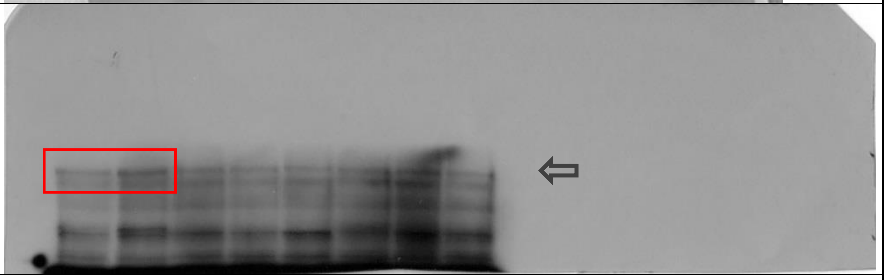
PTP1B (50kDa)	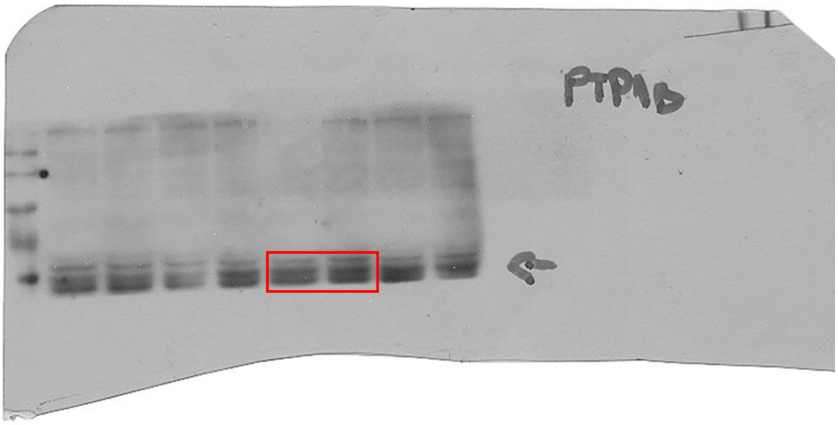

NFκB cytosols (65kDa)	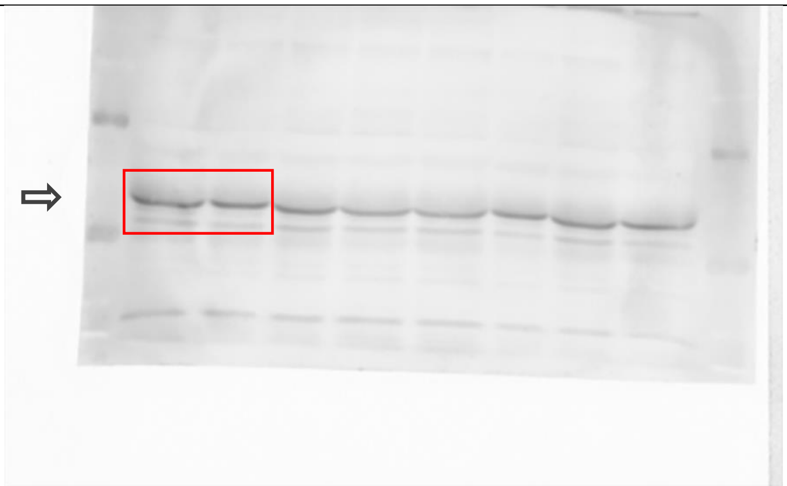
NFκB nucleosols (65kDa)	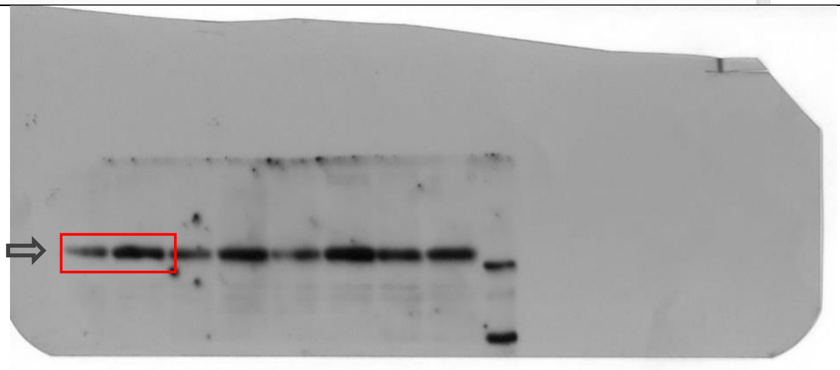
F4/80 (160kDa)	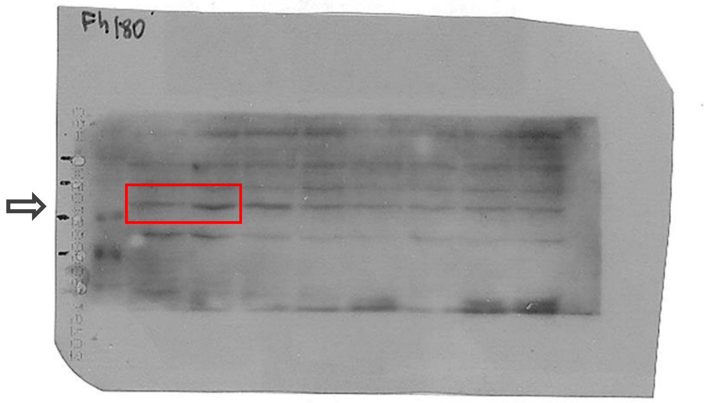
SOD1 (18kDa)	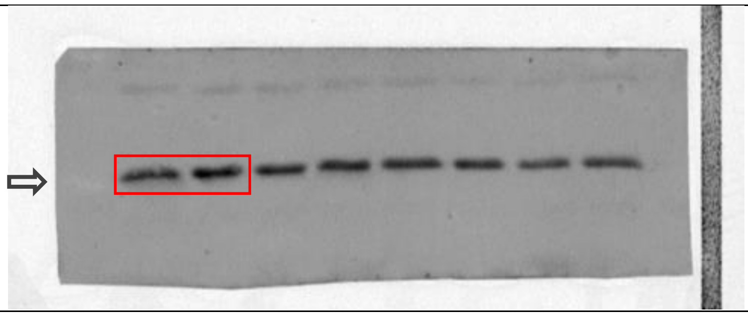

SOD2 (25kDa)	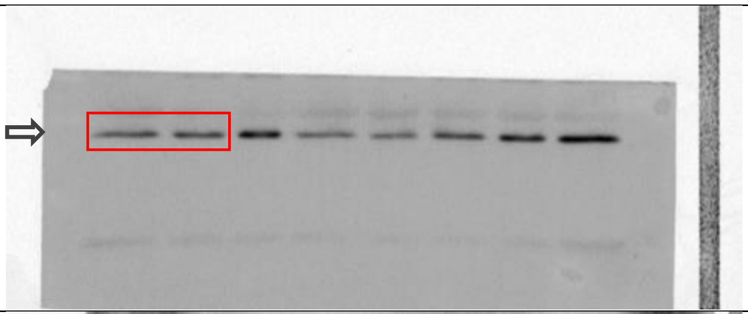
GRed (58kDa)	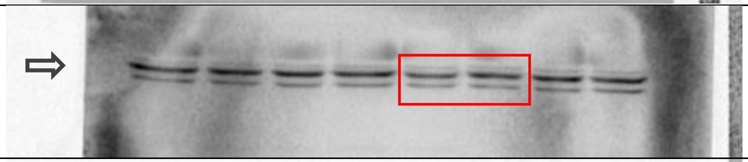
GPx (22kDa)	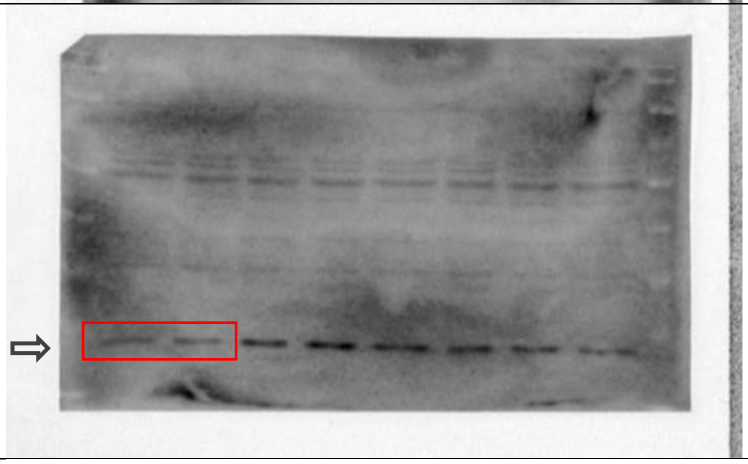
CAT (60kDa)	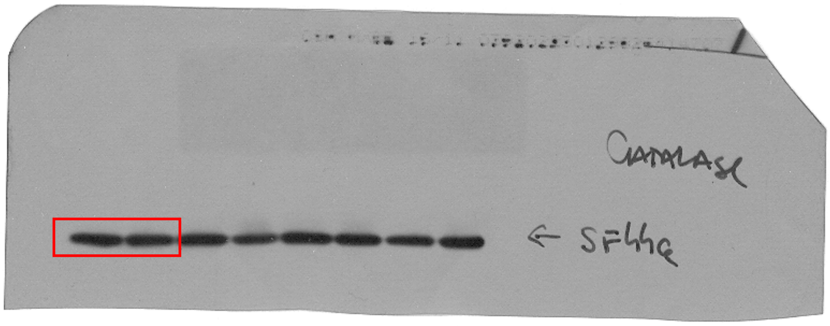
